# Prostate cancer-derived small extracellular vesicle proteins: the hope in diagnosis, prognosis, and therapeutics

**DOI:** 10.1186/s12951-023-02219-0

**Published:** 2023-12-14

**Authors:** Haotian Chen, Bairen Pang, Cheng Zhou, Meng Han, Jie Gong, Yong Li, Junhui Jiang

**Affiliations:** 1 Health Science Center, Ningbo University, Ningbo, 315211 Zhejiang People’s Republic of China; 2 Ningbo Clinical Research Center for Urological Disease, The First Affiliated Hospital of Ningbo University, Ningbo, 315010 Zhejiang People’s Republic of China; 3 Translational Research Laboratory for Urology, Department of Urology, The First Affiliated Hospital of Ningbo University, Ningbo, 315010 Zhejiang People’s Republic of China; 4 Cancer Care Centre, St George Hospital, Kogarah, NSW 2217 Australia; 5 School of Clinical Medicine, St. George and Sutherland Clinical Campuses, UNSW Sydney, Kensington, NSW 2052 Australia; 6 Department of Urology, Ningbo First Hospital, The First Affiliated Hospital of Ningbo University, Haishu District, Ningbo, 315600 Zhejiang People’s Republic of China

**Keywords:** Prostate cancer, Extracellular vesicle, Diagnosis, Prognosis, Metastasis, Therapeutics, Liquid biopsy

## Abstract

Current diagnostic tools for prostate cancer (PCa) diagnosis and risk stratification are insufficient. The hidden onset and poor efficacy of traditional therapies against metastatic PCa make this disease a heavy burden in global men’s health. Prostate cancer-derived extracellular vesicles (PCDEVs) have garnered attention in recent years due to their important role in communications in tumor microenvironment. Recent advancements have demonstrated PCDEVs proteins play an important role in PCa invasion, progression, metastasis, therapeutic resistance, and immune escape. In this review, we briefly discuss the applications of sEV proteins in PCa diagnosis and prognosis in liquid biopsy, focus on the roles of the PCa-derived small EVs (sEVs) proteins in tumor microenvironment associated with cancer progression, and explore the therapeutic potential of sEV proteins applied for future metastatic PCa therapy.

**Graphical Abstract:**

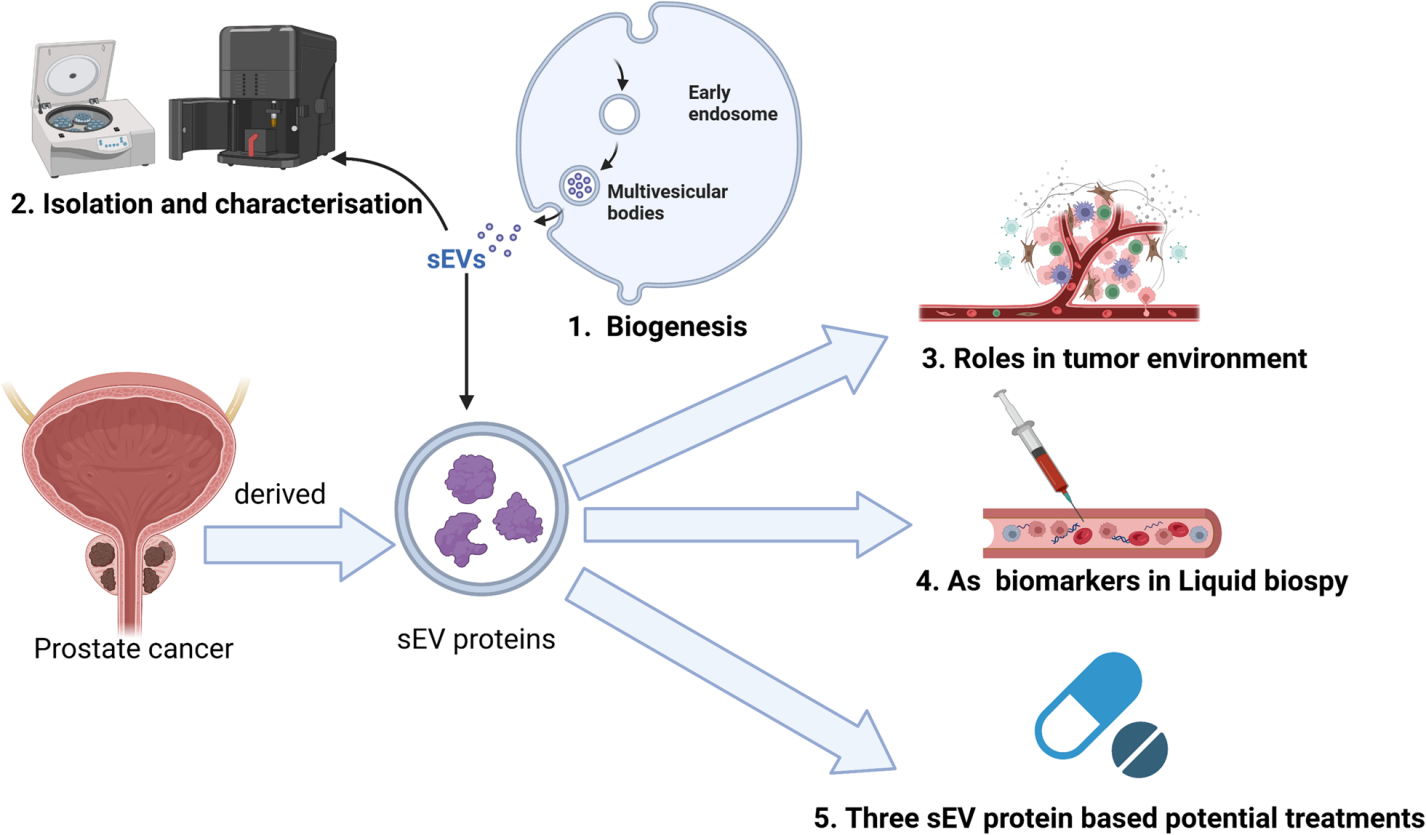

## Introduction

Prostate cancer (PCa) is the most common cancer in men worldwide. It was estimated that there would be 288,300 new cases of PCa in the United States in 2023, accounting for 29% of new cases of male cancers, with an estimated 34,700 deaths, accounting for 11% of male cancer deaths [1]. The incidence of PCa in Asia–pacific areas such as China is increasing with more mortality [2]. Tumor staging has a significant impact on treatment and prognosis. While generally, the non-metastatic localized PCa patients have a good prognosis, with a 5-year survival rate of 60–90%, distant metastatic PCa patients lead to irreversible disease progression, with a 5-year survival rate of only 30–40% [3]. The proportion of patients diagnosed with metastatic disease has witnessed a significant 25% increase in the past decade [4]. The elevated mortality rate is closely linked to the extensive spread of metastasis. Although surgical procedures and radiation therapy can effectively address localized disease, up to 30% of PCa patients treated in such a manner experience relapse and metastasis. Unfortunately, there is currently no curative therapy available for metastatic PCa, and the median survival period is approximately 1 year.

A major challenge in PCa clinical management lies in the limitations of current diagnostic tests, such as serum prostate specific antigen (PSA) testing, digital rectal examination, and histopathologic grading of tissues, to discern between indolent and aggressive disease [5]. The only existing screening biomarker, PSA, has been a subject of controversy as a screening assay due to its limitations, including false negatives and a high positive rate [6]. PSA cannot be used for early diagnosis and stratifying progression risk groups. It often results in over-diagnosis and over-treatment ranging from 20 to 42%, which can potentially cause more harm than good to patients. Furthermore, PSA has demonstrated little or no benefit in terms of PCa-specific survival [7, 8]. Therefore, improving the screening and diagnosis of PCa-especially metastatic PCa, and in-depth research on the mechanism of PCa metastasis are of great importance to design reasonable treatment plans, and improve the prognosis of patients. There is an urgent need to find biomarkers that can replace the current diagnostic approaches for more accurate diagnosis tools and monitoring PCa progression [9].

Liquid biopsy has recently emerged as an appealing non-invasive strategy to support early cancer diagnosis, selection for biopsy, active surveillance for low-risk cancer and post-treatment for recurrence. Liquid biopsy is a blood test that detects cancer cells or DNA or extracellular vesicles (EVs) that are circulating in the blood. It has demonstrated unparalleled advantages over conventional tissue biopsy and may complement or even replace medical imaging as a first- or second-line screening tool for earlier cancer detection and better surveillance of cancer metastasis and prognosis [10–12]. Liquid biopsy includes the analysis of circulating tumor cells (CTCs), circulating tumor DNA (ctDNA), and EVs [13, 14]. However, the utilization of CTCs or ctDNA as cancer biomarkers encounter various technical and translational challenges including difficult isolation and characterization, a short half-life, high fragmentation, low abundance, and low stability. EVs can be classified to different subtypes based on different biogenesis pathways (exosome, microvesicles), physical characteristics [small EVs (sEVs), large EVs (lEVs),)], biochemical composition (CD81^+^/syntenin^+^-EVs), or descriptions of origin (large oncosomes, podocyte EVs). EVs possess several advantages including abundance (10^8–13^ exosomes/mL in plasma), stability (capable of being stored at – 80 ℃ for months and even years), easy accessibility and having tumor-specific surface signatures.

**Fig. 1:**
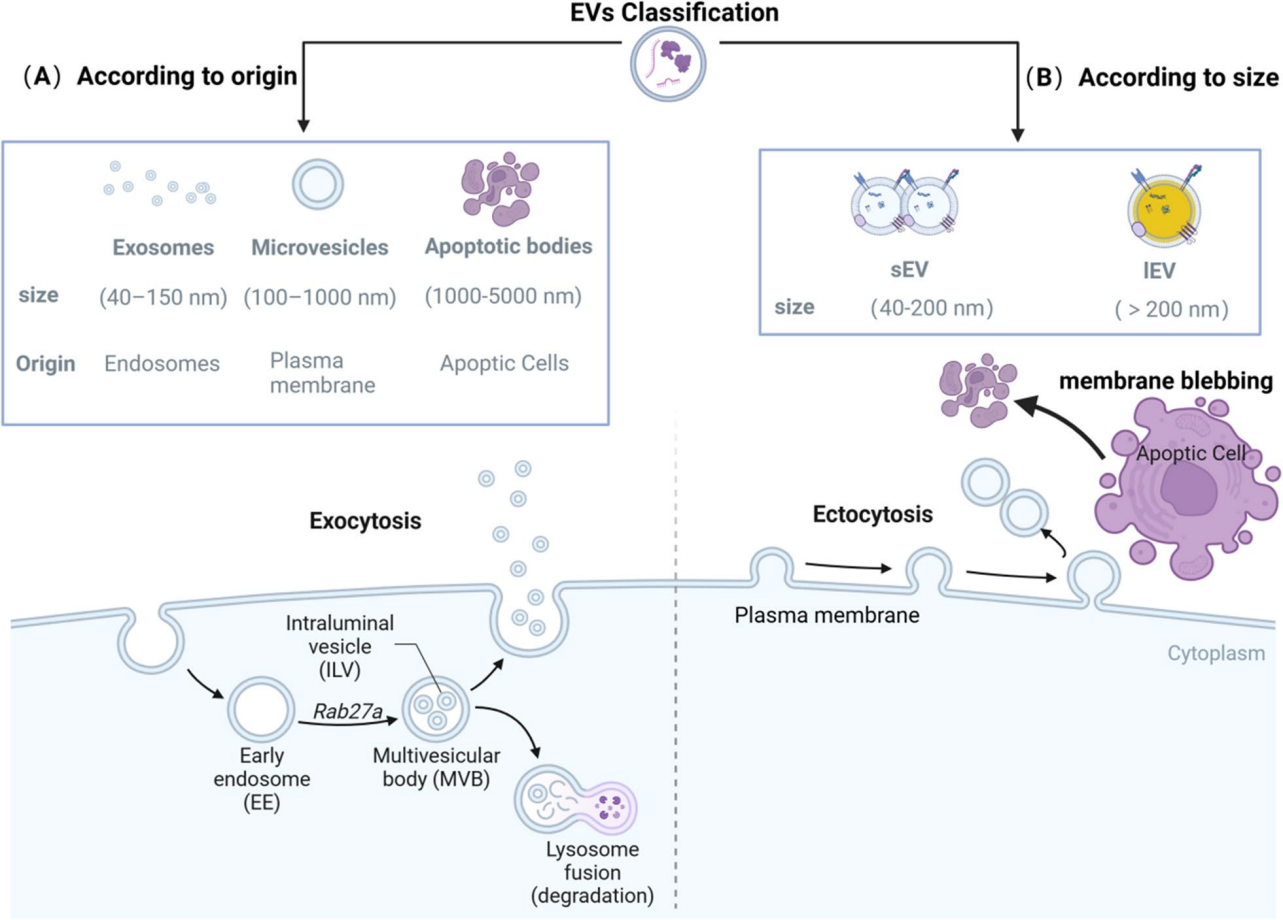
Two classifications of EVs. **A** According to the origin of EVs, they can be classified as exosomes, microvesicles and apoptotic bodies. Exosomes are formed by early endosome formation, MVB development, plasma membrane transportation fusion and exocytosis. Microvesicles are formed by ectocytosis of the plasma membrane and apoptotic bodies are formed directly by means of membrane blebbing of apoptotic cells. **B** According to the particle size, EVs are divided into sEVs and lEVs This figure was created with BioRender.com

All cells release EVs that are also classified into different types based on their morphology and contents, such as exosomes (sEVs), microvesicles (lEVs) and apoptotic bodies. Figure 1 describes the two classifications of EVs. Exosomes are released from all kinds of live cells after multivesicular body (MVB) fused with the cell membrane and can be detected through a range of methods, including high-resolution electron microscopy (EM) and mass spectrometry (MS) [15]. Exosomes biogenesis originates from the endocytic pathway, which involves several steps: (1) the plasma membrane buds inward to form early endosomes, (2) early endosomes develop into late endosomes and MVBs containing intraluminal vesicles (ILVs), (3) MVBs are transported to the plasma membrane, and (4) some MVBs are degraded after fusing with lysosomes, while others, which have specific surface proteins, are released in vesicular form after fusion with the plasma membrane, forming exosomes [16]. Multiple mechanisms are involved in each step, such as the endosomal sorting complex required for transport (ESCRT) or non-ESCRT mechanisms, which have been shown to depend on different MVBs to achieve the biogenesis of ILVs. ESCRT consists of four complexes: ESCRT-0, ESCRT-I, ESCRT-II, and ESCRT-III. ESCRT-0, made up of hepatocyte growth factor-regulated tyrosine kinase substrate (Hrs) and signal transducing adaptor molecule (STAM), recognizes mono- or polyubiquitinated cargo proteins through its ubiquitin-binding domain, while ESCRT-I and ESCRT-II complexes are responsible for inward budding of the endosomal membrane. The ESCRT-III complex then mediates the fusion of ILVs, ultimately generating ILVs. Research has shown that even if all four key subunits of the ESCRT complexes are simultaneously silenced, ILVs still form in MVBs, indicating the existence of ESCRT-independent mechanisms [17]. These mechanisms include two pathways: (1) Tetraspanins play a crucial role in clustering membrane cargos and localizing to specific membrane domains that facilitate the budding of ILVs; and (2) The recruitment of factors such as flotillins and the autophagy-related protein LC3 occurs in lipid domains that are enriched in ceramide and cholesterol [18]. Various molecules, including Pmel17 and RAB31, have been found to be involved in ESCRT-independent biogenesis [19, 20].

sEVs are nano-sized extracellular vesicles (40–200 nm) in diameter, one of the most common investigated EV subpopulations released by cells into biological fluids that contain a plethora of biomolecules such as proteins and nucleic acids [21, 22]. sEV may share some pathways mentioned above with exosomes. Due to their high representation of the cell of origin, sEVs serve as potent and clinically valuable tools for early diagnosis and prognosis of PCa [23]. Accumulating studies indicate that the levels of sEVs in body fluids have significant clinical relevance to cancer status [24, 25]. sEVs were reported to promote tumor growth by transporting their contents to recipient cells to regulate their cellular functions, and these biological active molecules have caught great attention in cancer research [26]. Compared to genetic information changes, the key regulatory proteins found in sEVs offer more direct information regarding disease progression. The communication of sEVs proteins plays a critical role in tumor progression and metastasis not only by regulating the tumor microenvironment (TME) around in situ tumor cells, but also by affecting distant metastatic sites and inducing the formation of pre-metastatic niches (PMN) [27]. This review discusses the biogenesis and contents of sEVs and summarizes the methods for isolation and characterization of sEVs. Furthermore, this article primarily focuses on the role of sEV proteins in the TME of PCa, as well as their application as biomarkers for PCa diagnosis and prognosis. Finally, the potential prospects of using sEV protein-based therapeutic approaches for PCa are also discussed.

## sEV isolation and characterization

Despite the significant potential of sEVs in cancer diagnosis, prognosis and therapeutics, the biggest challenge we face since their discovery is how to efficiently isolate and purify sEVs. So far, various methods have been developed for the separation of sEVs and the detection of sEV proteins and nucleic acids.

### Ultracentrifugation (UC)

UC is currently the most used separation method and includes differential ultracentrifugation and gradient density ultracentrifugation. Differential ultracentrifugation separates different extracellular components of the fluid sample according to their density, size, and shape under a certain centrifugal force. Due to its convenience, low technical requirements, and the ability to be used with large scale and without complex sample pre-treatment, differential ultracentrifugation has been widely regarded as the gold standard technology for the purification of sEVs for the past 30 years. However, the density gradient centrifugation can maximize the removal of interference caused by proteins. sEV samples prepared by differential ultracentrifugation often have low purity compared to density gradient ultracentrifugation [28]. Density gradient ultracentrifugation separates particles by layering them on different densities of biocompatible media (such as sucrose). Although sEVs obtained by density gradient ultracentrifugation have higher purity than those obtained by differential ultracentrifugation [29], this method requires large sample volumes, and is time-consuming, uses expensive equipment, and involves complex steps [30]. Currently, extensive research has successfully isolated sEVs from both PCa cells and the plasma of PCa patients using UC. These sEVs were utilized as a benchmark to evaluate the pros and cons of novel isolation techniques [31, 32]. One study employed UC to identify over 3000 sEV proteins extracted from urine samples of PCa patients. Significantly, the majority of these proteins exhibited the close links with sEVs, thus validating the reliability of EV extraction from urine samples [33]. Another study demonstrated that further refinement of UC methods significantly enhanced the enrichment of pertinent proteins in sEVs derived from urine samples of PCa patients, presenting an encouraging strategy for future investigations [34]. In a separate study, Dhondt et al. devised a separation strategy utilizing Optiprep density gradient centrifugation to enrich sEVs from urine samples of patients with benign prostatic hyperplasia (BPH) and PCa. Through this approach, they identified a total of 3686 proteins, including several hundred proteins not previously documented in known human urine sEV proteomes [35]. All these results suggest that UC is still a valuable standard tool for PCa EV isolation.

### Size-based methods

Ultrafiltration techniques and size exclusion chromatography (SEC) are two size-based methods. Ultrafiltration techniques are similar to traditional filtration methods. Ultrafiltration employs ultrafine nanomembranes with various critical molecular weight cut-off (MWCO) values to separate sEVs from clinical samples or cell culture media. This is a size-based method for sEV separation. Compared to UC, ultrafiltration is less time-consuming and doesn’t require special equipment. However, filtration-based methods easily have blockage issues, and unavoidable absorption on membrane would result in a loss of sEVs and possibility of false negative molecular result. SEC is a method used to separate biological molecules based on the size of the sample and the pore size of the gel. Smaller molecules briefly pause in the matrix, resulting in a longer elution time through the chromatography column, while larger molecules are eluted first. Unlike filtration and centrifugation methods, SEC is a gentler technique that can preserve the biophysical and bioactive characteristics of sEVs [36]. This method is commonly used in human plasma samples for downstream analysis. A study successfully isolated sEVs from normal prostate RWPE1 cells using ultrafiltration and characterized their associated proteins [37]. Another study found that SEC demonstrated advantages of high purity and low protein contamination in the extraction of sEVs from the plasma and urine of PCa patients, ensuring the quality requirements for subsequent downstream analysis [38, 39]. Chen et al. developed a highly efficient sEV isolation platform called Exosome detection via the ultrafast-isolation system (EXDUS) that is based on ultrafiltration, which achieves ultrafast and efficient purification of sEVs through negative pressure oscillation and double-coupled harmonic oscillator-enabled membrane vibration. EXDUS enables maximum enrichment of sEVs from different types and volumes of biological fluids by changing the oscillation mode. Currently, the application of EXDUS in urine samples from bladder cancer and kidney cancer patients within the urogenital system has demonstrated significantly higher yields and purities of isolated sEVs compared to other isolation techniques. Successful downstream studies have been conducted using EXDUS for the satisfied results, indicating promising prospects for its wide application in PCa research in the near future [40].

### Immunoaffinity capture

This method relies on specific immune interactions between target proteins (antigens) on the surface of sEVs and antibodies to obtain highly purified sEVs [41]. Immunoaffinity capture methods are an ideal platform for separating subsets of EVs with specific origins due to their ability to recognize specific biomarkers. This results in a high level of purity and selectivity. However, the high cost, low yield, and the need for additional elution steps limit their application [28]. The reliability of immunoaffinity technology has been substantiated in a PCa study. This method facilitates the high-purity isolation of sEV products from both PCa cells and patients’ plasma. Through continuous optimization of reagent selection and protocols, this method can further enhance the capture efficiency and sensitivity for downstream protein and RNA analysis. Immunoaffinity technology demonstrated applicability to complex and low-volume samples [42]. Moreover, a comparative investigation utilizing atomic force microscopy (AFM) and nanoscale flow cytometry (NFC) evaluated immunoaffinity approaches and commercial kits, indicating that immunoaffinity approaches more effectively eliminate the impact of plasma proteins on sEV purity in the plasma of PCa patients. This finding is very important in the pursuit of PCa-specific biomarkers, as it helps to minimize or diminish plasma protein interference in sEV products [43]. This approach holds promise in prostate cancer-derived extracellular vesicles (PCDEVs) biomarker research.

### Commercial kits

Precipitation method mainly relies on using polymers to precipitate sEVs, typically using polyethylene glycol (PEG) as a medium. After co-incubating sample with PEG solution at 4 ℃ overnight, sEVs are collected by low-speed centrifugation. This method is simple and doesn’t require special equipment, often producing high yields of sEVs suitable for processing large samples [44]. However, it was found that nucleic acids, protein contaminants, and some polymer residues inevitably appear in the products, resulting in lower purity and the potential for false positives [45]. Nevertheless, this method is a promising, inexpensive, and fast strategy for sEV isolation.

Several studies have conducted evaluations on the effectiveness of different methods for isolating sEVs from PCa cells and patient plasma. These studies have found that the purity of sEVs obtained through precipitation methods is consistently the lowest [38, 46]. Furthermore, precipitation methods have been widely applied in the exploration of diagnostic biomarkers for PCa. Multiple studies have identified the diagnostic value of sEVs prostate cancer specific antigen (PSMA) and lncRNA in the urine of PCa patients using precipitation methods [47, 48]. Mercadal et al. discovered that the miRNA expression profile of sEVs extracted from the semen of PCa patients using various isolation methods, including precipitation, exhibited variations. The possible reason for these variations is that different extraction methods yield different sEV subtypes. However, these differences have minimal impact on the performance of the proposed novel diagnostic model using combined semen sEV miRNA and blood PSA levels for improving PCa diagnosis [49]. Generally, commercial kits are applicable in cell line sEV isolation or plasma and urine isolation for sEV miRNA analysis in PCa research.

### Microfluidics

Microfluidics-based techniques are important tools for integrating the isolation and detection of sEV. They combine microfluidic technology with traditional isolation techniques and rely on the physical and chemical characteristics of sEVs, such as size, density, and surface antigens, to achieve rapid and high-purity separation. The resulting detection technology meets the requirements for high-throughput and high-precision [50]. However, this technology is still in the early stages of development and improvements are needed in the future. Microfluidics has garnered increasing attention in the realm of PCa research. A groundbreaking advancement comes in the form of a 3D Self-Assembled Nanostructured SiO2 Microfluidic Chip. This innovative chip facilitates the efficient separation and detection of sEVs from PCa cells and the plasma of PCa patients, while significantly reducing sample consumption compared to conventional methods. The chip has several notable advantages, including straightforward preparation, enhanced affinity between sEVs and the chip surface, and highly sensitive analysis. By utilizing the 3D-SiO2 porous chip equipped with three markers (CD81, EpCAM, PSMA), early diagnosis and risk stratification of PCa patients become feasible [51]. Additionally, a study has successfully developed an acoustic microfluidics-based technique for enriching sEVs from urine samples of PCa patients, demonstrating exceptional efficiency and continuous enrichment of sEVs. Notably, certain differentially expressed miRNAs identified in these sEVs potentially serve as biomarkers for PCa diagnosis [52]. This approach is promising and applicable in clinical settings due to its small sample volume, more sensitive and specific characteristics, portable and point of care for patients.

### Combination approach

Individual isolation methods often have their inevitable drawbacks. In recent years, some researchers have proposed combining two or more separation techniques. Currently, microfluidics-based techniques occupy a prominent position in combined separation techniques. In addition to combining with different traditional isolation methods, individual traditional isolation method is also combined with acoustic, microfluidic viscoelasticity, and other techniques, having shown promising results in some studies [50, 53]. Furthermore, one study found that the combination of UC and SEC (qEV column) was more effective in enriching sEVs than either method alone, resulting in improved yield and efficiency in animal plasma sample [54]. With the growing interest in sEVs, future research will undoubtedly require a large number of purer sEV samples for mechanism investigation and therapeutic purpose. Whether combined separation techniques can overcome limitations in sEV isolation and achieve or approach our ideal requirements remains to be further studied. In PCa-related study, it was discovered that combined approach demonstrated greater value in sEV isolation. The combination of ultrafiltration and SEC in the separation of PCa urine samples significantly reduced contamination from non-EV proteins and ensured the maximum purity of sEVs [55]. It is worthwhile testing different combination approaches in clinical samples such as blood and urine to obtain purer sEVs for downstream analysis in PCa biomarker research. Table 1 summarizes each isolation method along with its respective advantages and disadvantages.

**Table 1:** Summary of the current EV isolation methods with advantages and disadvantages

Isolation Method	Advantage	Disadvantage	References
UC	Low technical requirements, suitable for large scale, simple pre-treatment, no extra contaminations, suitable for downstream analysis	Requiring large sample volumes for high efficiency, time-consuming, high initial capital cost,	[28, 30–32]
Size-based methods	Less time-consuming, low equipment requirement,	Blockage issues, membrane absorption	[61, 62]
Immunoaffinity capture	High purity and selectivity, suitable for complex and low-volume samples	High cost, low yield, extra elution steps	[28, 42]
Commercial kits	Equipment friendly, high yields, suitable for large samples,	Lower purity, false positives	[44, 45]
Microfluidics	Rapid, high-purity separation, portable	Not suitable for large scale, need extra validation	[50, 63]
Combination approach	Improved yield, efficiency and purity	Need further study and validation	[54, 55]

### Other new approaches recently developed

In recent years, several research teams have been dedicated to developing alternative novel methods for sEV isolation, which have garnered significant attention due to their unique double-layer lipid structure. Sun et al. discovered a novel and effective technique for sEV enrichment. This method utilizes the lipid bilayer structure of sEVs to selectively label the lipid components with trans-cyclooctene (TCO). The labeled sEVs are subsequently immobilized onto tetrazine (Tz)-grafted microbeads through a bioorthogonal click chemistry reaction, enabling the straightforward separation and collection of the captured sEVs. This approach shares some similarities with immunocapture techniques. Immunocapture relies on the availability of specific antigen molecules, and this method effectively overcomes the limitations of immunocapture, demonstrating a higher sEV capture rate in cancer cells compared to UC, ExoQuick, and other methods. Notably, this method has shown promising results in the downstream mRNA analysis of sEVs isolated from plasma samples of patients with Ewing sarcoma or pancreatic cancer, suggesting its diagnostic and monitoring potential [56]. Another study focuses on phosphatidylserine (PS), a lipid molecule present in the lipid bilayer structure. By exploiting the calcium-dependent binding of PS to TIM4, a high-purity isolation of intact sEVs was achieved [57].

An innovative approach utilizing dip-pen nanolithography creates microscale arrays of lipid patches for sEV capture, ensuring maximal preservation of RNA components within sEVs for subsequent analysis. This method demonstrates strong sensitivity and specificity [58]. Field flow fractionation (FFF), a sEV separation technique based on charge, leverages the differential charges carried by sEV subtypes to achieve efficient and high-purity label-free separation [59]. Asymmetrical flow field-flow fractionation (AF4) in combination with capillary electrophoresis (CE) is another label-free sEV isolation method. AF4 separates sEVs based on size, while CE further distinguishes sEVs of the same size but with different charge characteristics from abundant matrix components such as lipoproteins. The combination of AF4 and CE enhances separation efficiency and holds great value in studying sEVs and their subgroups. However, the carrying capacity of CE is limited, and its effectiveness for continuous processing of large samples remains a subject of debate, thereby limiting its widespread clinical application [60]. The emergence of these novel technologies has provided deeper insights into the physicochemical properties of sEVs and holds the potential to become routine isolation methods with ongoing advancements in the field. These novel EV isolation approaches have a great chance to be used in PCa research in the future.

### Characterization of sEVs

**Fig. 2:**
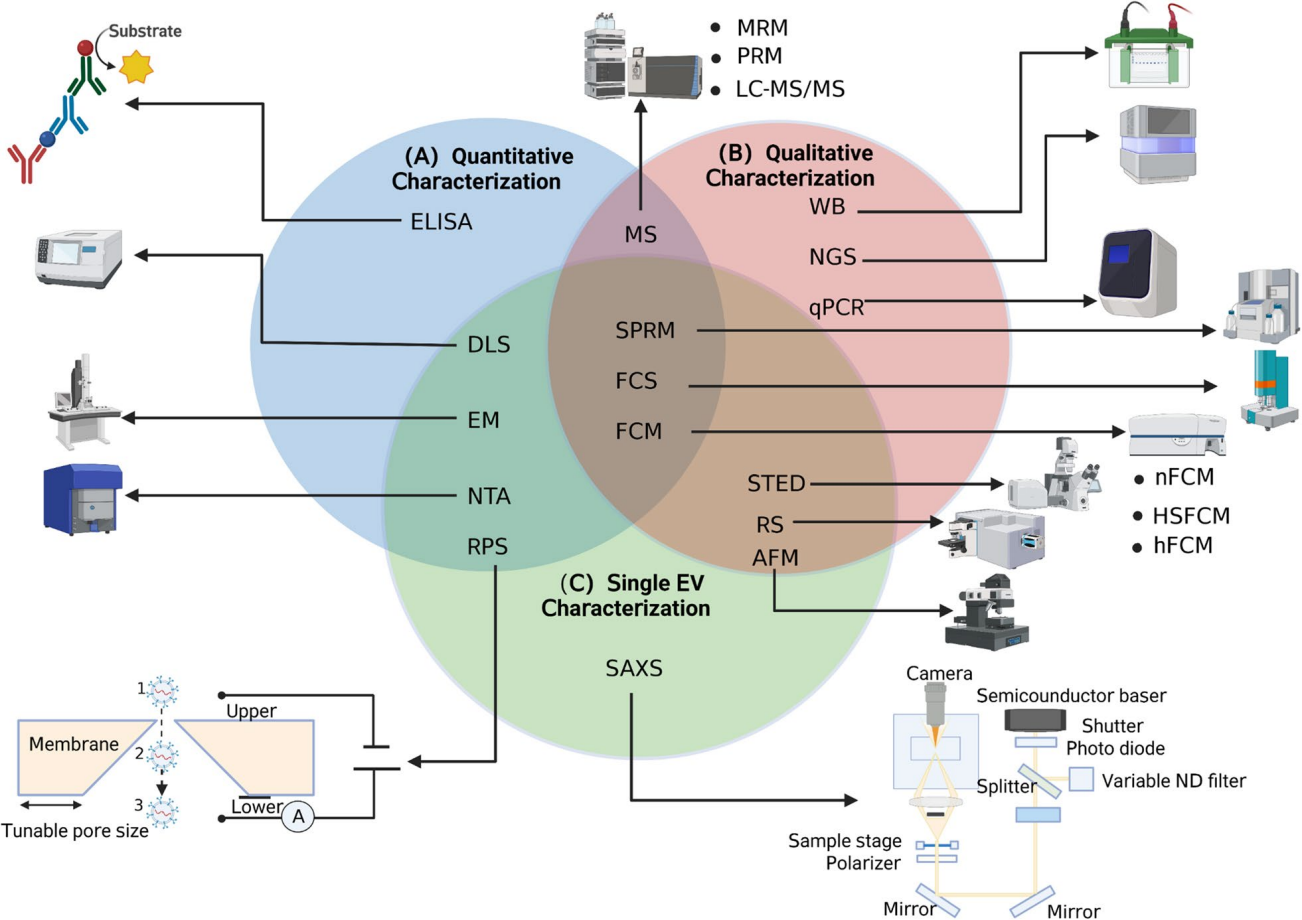
Small extracellular vesicle (sEV) quantitative, qualitative, and single EV characterizations. sEVs isolated from prostate cancer (PCa) blood or urine samples and cell lines can be characterized by size, morphology, concentration, and sEV markers. **A** quantitative characterization. This is performed by enzyme-linked immunosorbent assay (ELISA), dynamic light scattering (DLS), electron microscopy (EM), nanoparticle tracking analysis (NTA), resistive pulse sensing (RPS), multiple reaction monitoring (MRM), parallel reaction monitoring (PRM), liquid chromatography with tandem mass spectrometry (LC–MS/MS), surface plasmon resonance microscopy (SPRM), fluorescence correlation spectroscopy (FCS) and flow cytometry (FCM). **B** qualitative characterization. This is completed by Western blot (WB), next-generation sequencing (NGS), quantitative polymerase chain reaction (qPCR), MRM, PRM, LC–MS/MS, SPRM, FCS, FCM, stimulated emission depletion (STED) microscopy, RS and Atomic force microscopy (AFM). **C** single EV characterization. This is done by small-angle X-ray scattering (SAXS), DLS, EM, NTA, RPS, SPRM, FCS, FCM, Nano flow cytometry (nFCM), high-sensitivity flow cytometry (HSFCM), and high-resolution flow cytometry (hFCM), RS and AFM This figure was created with BioRender.com

sEVs possess the property mainly based on their distinctive characteristics such as their size, morphology, floatation density, and the presence of marker proteins. According to the Minimal Information for Studies of Extracellular Vesicles 2018 (MISEV2018) recommendations, EV identification includes western blot verification of EV-specific markers such as CD9, CD81, CD63, electron microscopy (EM) or nanoparticle tracking analysis (NTA) and at least two characterization methods needs to be performed [64]. The methods used to characterize sEVs are in line with their distinct features and can be broadly categorized into three types: quantitative, qualitative and single-vesicle characterization [65]. The summary of sEV characterizations applied in PCa is shown in Fig. 2. It is important to note that these classifications are intended to simplify the understanding and categorization of sEV characterization techniques, and are not strict definitions, as some techniques have overlapping features between quantitative characterization, qualitative characterization and single EV characterization. In practical applications, it is often necessary to combine multiple techniques to obtain comprehensive and accurate characterization results of sEVs.

#### Quantitative methods

Quantitative characterization is used to assess the success of sEV isolation and the quality of the products, including the yield and purity of biomolecules such as proteins, nucleic acids, and lipids. Techniques such as EM, NTA, flow cytometry (FCM), fluorescence correlation spectroscopy (FCS), dynamic light scattering (DLS), resistive pulse sensing (RPS) are primarily used to identify the total number of sEVs and quantify their yield by measuring the total number of particles within a certain size range [66]. Among these, NTA and FCS are the most used methods. Protein content is also an important indicator of purity, and enzyme-linked immunosorbent assay (ELISA) and mass spectrometry (MS) are representative techniques for quantifying it. In addition, surface plasmon resonance microscopy (SPRM) converts SPR response into surface binding mass for quantitative analysis of sEVs [67]. By combining with specific capture of target sEV proteins, it allows for the analysis of specific protein concentrations [68]. The ratio of protein content to total sEV particle count can help determine sample purity.

#### Qualitative methods

Qualitative methods are primarily employed to identify sEVs, validate proteomic and lipidomic identifications, and sequence coverage of DNA/RNA. Atomic Force Microscopy (AFM) is used to reveal the surface properties and structural features of sEVs for preliminary qualitative analysis [69]. Techniques such as Western blot (WB), FCM, stimulated emission depletion (STED) microscopy, and surface plasmon resonance microscopy (SPRM) are utilized to qualitatively characterize sEVs based on their protein markers [70]. Meanwhile, FCS labeled lipid-binding proteins, Raman spectroscopy (RS), and liquid chromatography with tandem mass spectrometry (LC–MS/MS) are used to identify lipid and protein markers, while multiple reaction monitoring (MRM), and parallel reaction monitoring (PRM) are used to validate identified peptides or lipids. Microarray technology, next-generation sequencing (NGS) and quantitative polymerase chain reaction (qPCR) are employed to analyze the genomic content of sEVs.

#### Single EV methods

The single EV characterization method focuses on individual sEV characteristics, including size, structure, and chemical composition. Atomic force microscopy (AFM), STED, SPRM, small-angle X-ray scattering (SAXS), and scanning electron microscopy (SEM) identify the typical cup-shaped structure of sEVs. NTA, DLS, EM, SAXS, FCM, and tunable resistive pulse sensing (TRPS) help identify the size of individual sEVs, while RS can identify their chemical composition. Nano flow cytometry (nFCM), High-sensitivity flow cytometry (HSFCM) and high-resolution flow cytometry (hFCM) are used to identify very small EVs and EV subpopulations.

These techniques help determine the purity and yield of products and choose the most appropriate separation method, as well as evaluate the therapeutic value of sEVs as drug carriers [71].

## Mechanisms of sEV proteins in tumor microenvironment and prostate cancer pathology advancements

**Fig. 3:**
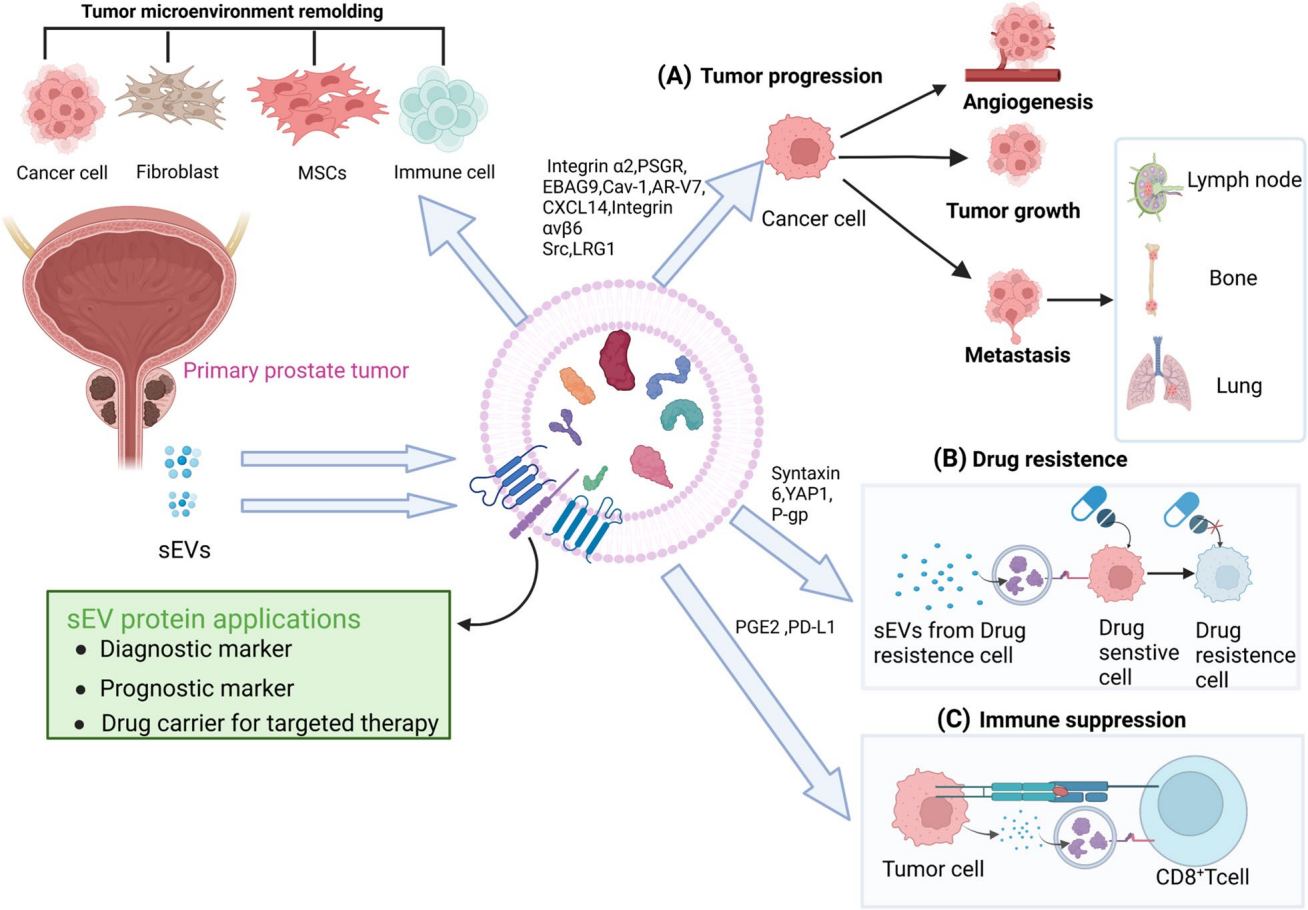
Overview of sEV proteins in prostate cancer angiogenesis, tumor growth, metastasis, drug resistance, immune suppression in the tumor microenvironment. **A** PCa cell-derived sEV proteins including Integrin α2, PSGR, EBAG9, Cav-1, AR-V7, CXCL14, Integrin αvβ6, Src, LRG1 promote angiogenesis, tumor growth, and metastasis in tumor cells, thereby accelerating tumor progression. **B** PCa cell-derived sEV proteins including Syntaxin 6, YAP1, P-gp converts drug sensitive PCa cells to drug resistant PCa cells, leading to the development of drug resistance. **C** PCa cell-derived sEV proteins including PGE2 and PD-L1 mediate the communication with CD8^+^ T cells, thereby participating in immune suppression. *AR-V7* androgen receptor-v7, *Cav-1* caveolin-1, *CXCL4* Chemokine (C-X-C motif) ligand 14, *EBAG9* estrogen receptor-binding fragment-associated antigen 9, *LRG1* leucine-rich alpha-2-glycoprotein 1, *MSCs* mesenchymal stem cells, *PCa* prostate cancer, *PSGR* Prostate-specific G-protein coupled receptor, *YAP1* Yes-associated protein 1, *P-gp* P-Glycoprotein, *PGE2* Prostaglandin E2, *PD-L1* programmed cell death ligand 1. This figure was created with BioRender.com

Increasing evidence has demonstrated that PCa cells release a higher amount of sEVs compared to normal cells [72]. sEV concentrations were increased as PCa progressed from low-grade to high-grade [73], indicating PCa-derived sEVs play an important role in cancer progression and metastasis. These PCa-derived sEVs may play a significant role in regulating communication between tumor cells, the tumor microenvironment (TME), and distant metastasis sites through both autocrine and paracrine mechanisms [74]. This promotes signal transduction and the formation of pre-metastasis niches (PMNs), ultimately contributing to tumor development and metastasis [75]. Notably, cancer cells require sufficient oxygen to grow, and studies indicate that cancer-derived sEVs serve as carriers of various proteins, RNAs and lipids, which re-program the secretion of growth factors and cytokines by endothelial cells, activate signaling pathways, and prompt perivascular cell migration and the formation of new blood vessels [76]. Such conditions create a favorable circumstance for cancer invasion and metastasis [77]. Furthermore, cancer cell-derived sEV proteins predominantly inhibit anti-cancer immune responses by impacting T cells, dendritic cells (DCs), NK cells, macrophages, myeloid-derived suppressor cells (MDSCs), and regulatory B cells, thus facilitating immune evasion [78]. Accumulated studies reveal that cancer-derived sEV proteins mediate therapeutic resistance in cancer cells through various mechanisms, such as pumping anti-cancer drugs out of cells, promoting epithelial-mesenchymal transition (EMT), and regulating signaling molecules to promote anti-apoptotic pathways [79, 80]. Additionally, due to their high abundance, strong stability, and specificity, cancer-derived sEV proteins can serve as promising biomarkers for diagnosis and prognosis in liquid biopsy. Overall, cancer-derived sEV proteins play critical roles in facilitating carcinogenesis, angiogenesis, and drug resistance, and enable cancer cells to evade the host immune system, and act as valuable diagnostic and/or prognostic biomarkers as shown in Fig. 3. In this section, we focus on discussing the roles and applications of sEV proteins in PCa TME.

### sEV proteins promote prostate cancer growth and metastasis

#### sEV proteins induce EMT in prostate cancer

Cancer progression is a dynamic and multistep process, and sEV proteins play a significant role in the development of PCa by regulating the physiological functions of surrounding cancer cells and TME. EMT is a process whereby epithelial cells transition into a mesenchymal stem cell state, and in cancer, EMT is linked to tumor occurrence, invasion, metastasis, and resistance to therapy [81]. Recent studies have investigated the role of sEV proteins in EMT in PCa. For instance, the sEV-mediated integrin α2 subunit enhances the activity of Vimentin, FAK, and ERK1/2 in PCa cells, inducing EMT and ultimately promoting the development of PCa towards a more invasive form [82]. sEVs carrying Prostate-specific G-protein coupled receptor **(**PSGR) were found to induce low-invasive PCa cells to complete EMT, leading to a more invasive and metastatic phenotype and knocking down PSGR inhibited tumor cell proliferation and cloning [83]. Tumor-derived sEV-mediated EBAG9 protein is a critical factor that promotes EMT of PCa cells while inhibiting cytotoxic T cells, thereby facilitating tumor progression [84]. Additionally, PCa-derived sEVs containing Cav-1 were reported to promote EMT in NEPC via the NF-κB signaling pathway [85].

#### sEV proteins promote prostate cancer proliferation

The proliferation of cancer cells is a major driving force behind tumor progression. Fast-dividing cancer cells require a high level of energy to sustain their growth, which triggers the transition from oxidative phosphorylation to glycolysis. Intercellular communication between cancer cells and surrounding normal cells involves energy-requiring processes such as internalization, making this communication process particularly important. Recent research has shown that sEVs secreted by normal prostate epithelial cells and PC3 PCa cells exhibit inconsistent ATPase activity. The ATPase activity of PC3 sEVs is low comparing to that in normal prostate cells, resulting in the more ATP production, which increases intercellular communication and promotes tumor cell proliferation [86]. Moreover, sEV AR-V7 was shown to positively regulate AR signaling, promoting the proliferation of PCa cells, and affecting tumor growth [87]. sEV protein CXCL14 was found to promote M2 macrophage polarization through the NF-κB signaling pathway, thereby facilitating EMT in PCa cells. Its downregulation inhibited the proliferation and invasion of PCa cells, but did not affect apoptosis in PCa cells [88]. Studies have also shown that sEVs from adipocytes induced glycolysis and increased the growth rate of PCa cells through the activation of Akt and subsequent stabilization of hypoxia inducible factor-1α (HIF-1α) [89]. Additionally, a study has identified 1474 proteins in seminal sEVs that overlap with the PCDEVs database. Some of these proteins are involved in biological processes such as metabolic reprogramming, energy pathways, cell growth and maintenance, and transport, laying the foundation for further screening of their role in PCa growth [90]. Apoptosis and cellular senescence are crucial signaling pathways regulated by sEV proteins in PCa. Apoptosis is the major form of cell death in physiological conditions while senescence results in permanent cell cycle arrest. Both pathways serve as obstacles to tumor development [91]. A recent study revealed that sEV miR-143, derived from MSCs, inhibits the expression of TFF3, thereby suppressing the proliferation of PCa cells and promoting apoptosis [92]. Additionally, cellular senescence is defined as a stable cell cycle arrest in the G1 phase, providing a mechanism to inhibit cancer cell growth. Research has found that PCa cells release an increased amount of sEVs through associated mechanisms during treatment-induced cellular senescence, which transfers protein and genetic information between cells, indicating a certain connection between sEVs and cell apoptosis [93]. Further study has found that sEVs isolated from mock-treated senescent human lung epithelial carcinoma (A549) cells contain functional PTEN, and their transfer to PTEN-deficient PC3 cells leads to growth arrest [94]. However, the interaction between cellular aging and apoptosis suggests that inhibiting cellular aging to slow down cell growth may not always suppress tumor growth. Senescent cells exhibit enhanced metabolic activity, which may potentially contribute to tumor progression and recurrence. Moreover, if the immune system fails to clear senescent cells, or cancer cells escape senescence, which may become resistant to apoptosis stimuli [91].

#### sEV proteins associate with prostate cancer angiogenesis

Balancing the interaction between angiogenesis and tumor growth in the future is a challenge we must face. Angiogenesis is a multi-step process in which tumors form a new vascular system that is crucial for their growth [95]. Studies have shown that sEV αvβ6 integrin can regulate the level of survivin, increase the negative regulatory factor of angiogenesis pSTAT1, and promote PCa angiogenesis [96]. Additionally, it was found that the sEV Src protein in PCa cells activates focal adhesion kinase (FAK) through integrins, leading to angiogenesis and metastasis [97]. Moreover, high expression of α2-glycoprotein 1 (LRG1) was detected in the plasma sEVs of CRPC patients, further confirming sEV proteins play an important role in promoting angiogenesis and are associated with PCa progression [98]. These findings suggest that sEV proteins are a key player in promoting angiogenesis and the development of PCa.

#### sEV proteins influence prostate cancer fibroblasts and ECM remodeling

In the TME of PCa, the extracellular matrix (ECM) plays a crucial but often overlooked role. Remodeling of the ECM significantly impacts tumor cell proliferation and metastasis. In a DU145 and fibroblast co-culture model, the dynamic interaction between cancer cells and fibroblasts leads to pronounced changes in the ECM, thereby facilitating the invasive potential of DU145 cells [99]. During the progression of PCa, the prostate stroma undergoes phenotypic alterations, gradually transforming into cancer-associated fibroblasts (CAFs). This transformation triggers ECM remodeling, ultimately enhancing the invasive and progressive capabilities of PCa [100]. Consequently, CAFs assume a central role in the TME, gradually reshaping the biological functions of cancer cells through frequent communication. Recent research has shed light on the pivotal role of sEV TGFβ1 in driving fibroblast differentiation into myofibroblast-like cells, thus promoting the formation of a tumor-supportive stromal phenotype. These findings underscore the indispensable contribution of sEV TGFβ1 to the development of a pro-tumorigenic ECM [101]. Moreover, investigations have demonstrated that treating primary rat prostate fibroblasts with sEVs derived from metastatic PCa, as opposed to those derived from indolent non-metastatic PCa, significantly upregulates the mRNA expression of growth factors and cytokines, indicating fibroblast activation [102]. Furthermore, treatment of prostate fibroblasts with sEVs isolated from urine samples of PCa patients and healthy individuals elicits distinct transcriptional responses, whereas the transcriptional response in foreskin fibroblasts exhibits remarkable similarity. These findings provide further evidence that sEV derived from PCa patients exert a profound influence on the phenotypic and functional changes of fibroblasts [103]. There are reasons to speculate that sEV proteins of PCa cells play a significant role in this process.

#### sEV proteins associate with inflammation and immune escape in prostate cancer

The inflammasome is an important component of the TME, and various subsets of cells in the TME activate the inflammasome to regulate the progression of malignant tumors. Currently, researchers generally believe that the role of the TME in promoting/inhibiting tumor progression mainly depends on the type of tumor and inflammasome [104]. Inflammation is also a significant risk factor in the development of PCa. For example, the inflammasome NLRP3 was shown to enhance the proliferation and migration ability of PCa cells by activating caspase-1, thereby promoting the malignant progression of PCa [105]. Further studies have found that PC3-derived sEV induced the activation of NLRP3 inflammasome and caspase-1 through the ERK1/2 pathway, thereby creating a PCa inflammatory microenvironment [106]. Recently, it was discovered that through proteomic analysis and subsequent Ingenuity pathway analysis (IPA) of the sEVs derived from PCa cells of African American individuals, it was found that proteins loaded in sEVs correlate strongly with acute inflammatory response signalling pathways. In addition, Filamin A is closely associated with the occurrence of this inflammatory response. However, further investigation is still needed to elucidate the specific role of Filamin A in the transmission of inflammatory pathways [107].

Cancer cells invade various organs and tissues and spread to the body through metastasis, leading patients to an irreversible terminal stage of the disease. This process involves multiple mechanisms and steps [27]. In PCa, the primary sites of metastasis are the lymph node, bone, lungs, liver, and brain. The selection of metastatic sites by tumor cells is highly correlated with integrin avβ6, which is found in PCa-derived sEVs [108]. Further study shown that sEV integrin avβ6 inhibited the STAT1/MX1/2 signal transduction and reprogramed mononuclear cells into M2 tumor-supportive phenotypes, promoting the progression of PCa to the CRPC phenotype during metastasis [109]. The sEV protein PLD2 derived from C4-2B cells was reported not only to stimulate sEV secretion but also increase osteoblast activity, thus participating in the communication between PCa cells and the bone metastatic microenvironment [110]. PMN formation refers to the communication between tumor cells and cells in distant pre-metastatic organs, which helps to establish a tumor environment conducive to invasion, immune escape, and metastasis formation. sEVs serve as an effective medium in this process. The regulation of matrix metalloproteinases (MMPs) plays a crucial role in cancer metastasis [111]. Studies have found that under hypoxic conditions, sEVs secreted by PCa cells enhance the activity of MMPs in the hypothesized metastatic site, thereby reshaping the PMN [112]. Furthermore, it has been reported that the cholesterol homeostasis in bone marrow cells affects the reception and transduction of premetastatic signals mediated by sEVs released by PCa cells [113]. In recent years, there has also been a growing recognition of the role of sEV proteins in the PMN of PCa. A recent study has shown that sEVs released from C4-2B PCa cells stimulate the formation of PMN via the HIF1α-dependent pathway, which is mediated by pyruvate kinase M2 (PKM2) [114]. Furthermore, the EMT process is also a key factor in tumor cell metastasis, with the current consensus being that EMT is required during local invasion while the mesenchymal-epithelial transformation (MET) process is necessary during distant metastasis [115]. Previous studies demonstrated that the sEV protein Tspan8 in breast cancer cells plays a role in facilitating the progression of metastasis by mediating phenotypic changes in MET [116]. PCa and breast cancer share some similarities in inducing MET [117], Further research is still needed to support the involvement of sEV proteins in mediating MET occurrence in PCa.

#### sEVs promote pre-metastatic niche formation in prostate cancer

Although PCa bone metastasis is predominantly associated with osteoblastic changes, studies have found that osteoclasts still play a crucial role in PCa bone metastasis. One study revealed that sEVs derived from PCa cells are essential mediators in maintaining bone homeostasis. These sEVs promote osteoclast differentiation and inhibit the formation of osteoblasts in vivo and in vitro, thus creating a pre-metastatic osteolytic niche for PCa metastasis [118]. A recent study by Urabe et al. confirmed this finding, demonstrating that the protein CDCP1 on sEVs derived from metastatic PCa cells promotes the formation of mature osteoclasts. Furthermore, compared to localized PCa patients, CDCP1 showed high expression on plasma sEVs in patients with bone metastasis [119]. These results provide new insights for elucidating the mechanisms of osteoclast involvement in bone metastasis and identifying potential protein biomarkers of PCa bone metastasis for monitoring progression and evaluating treatment effects.

**Fig. 4:**
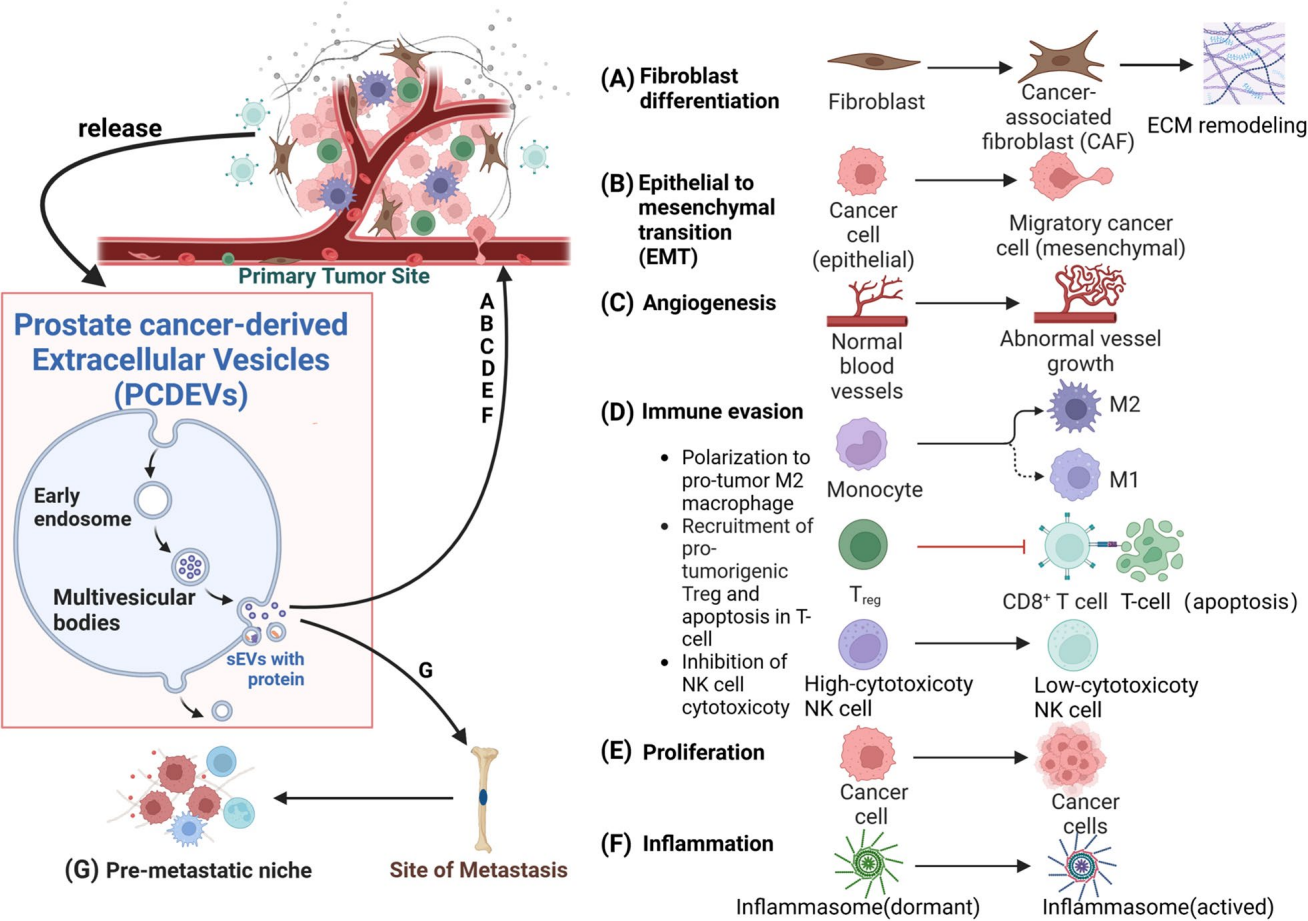
Outline of the alterations in each component regulated by sEV proteins in the PCa TME. In addition to exerting pro-tumorigenic effects at the primary site of PCa, sEVs proteins also play a role in creating conditions for metastasis at distant sites. **A** sEV proteins promote the differentiation of fibroblasts into cancer-associated fibroblasts (CAFs) and remodel the extracellular matrix (ECM). **B** sEV proteins promote epithelial-mesenchymal transition (EMT) in PCa cells. **C** sEV proteins facilitate abnormal angiogenesis, providing nutrients for tumor growth. **D** sEV proteins promote the transformation of macrophages into the M2 phenotype, recruit pro-tumorigenic regulatory T cells (Tregs), induce CD8^+^ T cell apoptosis, and inhibit natural killer (NK) cell cytotoxicity, thus facilitating immune evasion. **E** sEV proteins enhance PCa cell proliferation. **F** sEV proteins promote the activation of inflammasomes, leading to the occurrence of inflammation in the tumor microenvironment (TME). **G** sEV proteins act on distant sites to establish pre-metastatic niches (PMNs) This figure was created with BioRender.com

Overall, the progression and metastasis of PCa rely on the formation of the TME, wherein sEV proteins mediate cellular communication between cancer cells and host cells, influencing the phenotype or function of immune cells, fibroblasts, epithelial cells, inflammasomes, and endothelial cells. Figure 4 illustrates the alterations in each component regulated by sEV proteins in the PCa TME, including the polarization of CAFs, EMT, angiogenesis, disruption of immune homeostasis leading to an immune-evading TME, the promotion of cancer cell proliferation and invasion, proliferation, accelerated activation of inflammasomes, and establishment of a PMN in distant sites.

### sEV proteins in PCa drug resistance

Drug resistance is a major challenge in cancer treatment, and sEV proteins play a significant role in cell communication during the drug resistance process of cancer cells. The molecular mechanisms by which sEV mediate cancer resistance are generally categorized into three groups: (1) cancer cells excrete chemotherapeutic drugs through sEVs, (2) sEVs carry drug-resistant cargo and communicate with drug-sensitive tumor cells, and (3) sEVs act as bait targets in therapeutic drug [120]. Accumulating data shown that the mechanism of PCa drug resistance primarily belongs to the second category, where sEV proteins mediate intercellular communication between drug-resistant and drug-sensitive cells, modifying gene expression in the drug-sensitive cell population, enabling them to resist apoptosis when exposed to drugs [121]. One recent report suggested that sEVs mediated enzalutamide resistance (EnzaR) and treatment induced PCa neuroendocrine differentiation in PCa cells, and inhibiting sEV release partially restored the sensitivity of EnzaR PCa cells [122]. Another study also demonstrated the involvement of sEV release in the mechanism of EnzaR PCa. The inhibition of sEVs significantly suppressed the survival ability of resistant cells, but further research support is needed to determine whether sEV proteins are involved in the development of resistance [123]. In addition, increased expression of YAP1 was observed in sEVs isolated from EnzaR PCa patients’ serum, revealing that EnzaR sEVs enhanced LNCaP cell resistance by increasing YAP1 function [124]. Kharaziha et al. discovered that docetaxel-resistant DU145 cells release more sEVs than docetaxel-sensitive DU145 cells. Additionally, they identified a group of enriched proteins in the sEVs secreted by docetaxel-resistant DU145 cells [125]. sEV proteins also play a role in chemoresistance, as PCa cells carrying Cav-1 in metastatic CRPC (mCRPC)-derived sEVs acquire resistance to radiotherapy and docetaxel treatment [85]. Corcoran et al. reported that docetaxel-sensitive PCa cells (DU145, 22Rv1, and LNCaP) became resistant to docetaxel after exposure to docetaxel-resistant DU145 and 22Rv1 variants (DU145RD and 22Rv1RD), possibly due to the release of MDR-1/P-gp transporter proteins in sEVs. Similarly, Kato et al. found that the sEV p-gp level in docetaxel-resistant PC3 cells was higher than that in PC3 cells. Furthermore, downregulation of sEV p-gp reduced PC3 cell resistance to docetaxel, indicating that sEV-mediated protein transport plays a critical role in the development of PCa cell resistance [126]. Although the specific mechanism by which sEV proteins contribute to drug resistance in PCa remains unclear, inhibiting their formation and release may provide a new therapeutic strategy for treating this disease. Furthermore, targeting the PCa-derived sEV proteins through precision medicine may offer to overcome drug resistance and improve the current therapy efficacy. The role of sEV proteins in regulation of PCa drug resistance in TME is shown in Fig. 3.

### sEV proteins modulate immune escape during PCa progression

Cancer-derived sEV proteins impact the function and composition of immune cells within TME. Studies have shown that cancer-derived sEVs inhibited innate immune responses by mobilizing MDSCs, regulating/activating tumor-associated macrophages (TAMs), and neutrophils [127]. There is also direct evidence supporting the involvement of sEVs in immune evasion by PCa cells. For instance, sEVs derived from PCas were found to weaken the cytotoxic function of NK cells and CD8^+^ T cells by downregulating the activating receptor NKG2D, thus promoting cancer immune escape [128]. One recent report further demonstrated that avβ6 integrin in PCa is essential for monocytes to differentiate into M2-type immune suppressive cells. Interestingly, in another study, inhibiting Rab27a to block sEV release on DU145 cells significantly enhanced the tumor antigen-specific T cell response in dendritic cells (DCs). sEVs carrying PGE2 was found to induce the CD73 expression in DCs, resulting in T cell dysfunction in an adenosine-dependent manner [129]. Additionally, MDSCs are an important immunosuppressive cell type that mediates immune escape in the TME. sEVs derived from PCa cells were found to upregulate chemokine receptor 4 (CXCR4) by activating the TLR2/NF-κB signaling pathway, promoting the migration of MDSCs to the TME, and disrupting the balance of immune suppression [130]. The high expression of programmed cell death-ligand 1 (PD-L1) in PCa has recently received considerable attention, leading to the development of immune checkpoint inhibitors. The latest research has found that PD-L1 is also expressed on sEVs in some cancers, and inhibiting PD-L1 on sEVs through the Ca2^+^ channel ORAI1 induced anti-cancer responses [131]. In PCa, when PD-L1 binds to programmed cell death protein-1 (PD-1) on T cells, the anti-cancer activity of T cells was suppressed, and apoptosis was enhanced [132]. As PCa is a “cold” tumor, the ability of immunotherapy for inducing immune response is low compared with other “hot” tumors such as melanoma, lung cancer, breast cancer. So, it will be important and interesting to investigate sEV proteins as a therapeutic target for developing new immunotherapy to overcome immune escape in PCa patients, especially in CRPC and bone metastasis. The role of sEV proteins in the regulation of PCa immune escape is shown in Figs. 3 and 4**.**

## sEV proteins as diagnostic or prognostic biomarkers in PCa liquid biopsy

Liquid biopsy is a comprehensive and non-invasive diagnostic tool that retrieves tumor-related biomarkers from biological fluids, mainly including CTCs, ctDNA and EVs. Liquid biopsy plays a significant role in early diagnosis, risk stratification, residual monitoring, and recurrence of cancer. Among the three types of biomarkers, sEVs have gradually become a more widely used platform due to their numerous advantages. The dynamic variability of the mechanisms determines the heterogeneity of sEVs, which communicate between cells by carrying various contents such as proteins, lipids, and nucleic acids (mRNA, LncRNA, microRNAs, and DNA) [133]. The Exocarta database has so far identified 9769 proteins, 3408 mRNAs, 2838 miRNAs, and 1116 lipids in sEVs, and the content and type of these materials vary in different cell lines, indicating their complexity and potential functional diversity [134]. sEVs exhibit high heterogeneity, which is manifested in their size, content, function, and cell origin. Some sEV proteins display cell and tissue specificity, providing a basis for the study of related disease markers. Additionally, some surface proteins serve as sEV markers and play an important role in biogenesis. Table 2 summarizes the sEV protein markers related to PCa and discovered in the last 5 years for their source, application, and function, which will be discussed in the following.

**Table 2:** Summary of PCa-related sEV proteins in source, applications, and function

Biomarker	Source	Technology	Application	Function	References
TSP1	Cells	LC–MS/MS	Diagnosis	Upregulated in PCa	[135]
PKM2	Cells	WB	Therapy	Contributes to PCa metastasis to the bone	[114]
ACTN4	Cells/Plasma/ Serum	LC–MS/MS, WB	Prognosis/Therapy	Contributes to PCa progression and invasion	[136]
YAP1	Cells	WB	Therapy	Upregulated in EnzaR-PCa cells	[124]
MDR-1, MDR-3, Endophilin-A4 and PABP145	Cells/Serum	WB, nLC-MS/MS	Therapy	Upregulated in EnzaR-DU145 cells	[125]
CLD3	Plasma	LC–MS/MS, WB	Diagnosis	Upregulated in PCa	[137]
PSMA	Plasma	ELISA	Diagnosis	Upregulated in PCa	[47, 138]
PD-L1	Plasma	WB	Prognosis	Upregulated in Ra-233 treated patients with shorter OS	[139]
STEAP1	Plasma	FCM, WB	Diagnosis	Upregulated in PCa	[140]
CA IX	Plasma	WB, ELISA, enzyme activity assay	Diagnosis	Upregulated in PCa	[141]
CDCP1	Plasma	WB	Diagnosis	Upregulated in bone metastasis PCa	[119]
Integrin αvβ3	Plasma/Serum	WB	Prognosis	Promotes PCa aggressive phenotype	[142]
Del-1	Serum	ELISA	Prognosis	Upregulated in PCa	[143]
EphrinA2	Serum	WB, ELISA,	Diagnosis	Upregulated in PCa	[144]
Gammaglutamyl Transferase 1	Serum/Tissue	WB, ELISA, IHC	Diagnosis	Upregulated in PCa	[145]
FABP5	Urine	WB, LC–MS/MS	Diagnosis/ Prognosis	Upregulated in PCa	[146]
SERPINA3, LRG1, and SCGB3A1	Urine	DIA-MS	Diagnosis	Upregulated in PCa	[147]

*DIA-MS* Data Independent Acquisition Mass Spectrometry, *EnzaR* enzalutamide resistance, *ELISA* enzyme-linked immunosorbent assay, *FCM* flow cytometry, *IHC* immunohistochemistry, *LC–MS/MS* liquid chromatography with tandem mass spectrometry, *PCa* prostate cancer, *OS* overall survival

Recently, potential sEV protein biomarkers have been identified in both blood and urine in PCa patients (Table 2). These biomarkers have demonstrated a better performance and accuracy in determining cancer progression compared with traditional cancer markers such as prostate specific antigen (PSA). For instance, using MS-based proteomics, Bhagirath et al. discovered that sEV protein TSP1 is a highly sensitive and non-invasive biomarker for diagnosing neuroendocrine prostate cancer (NEPC) [135]. Additionally, another report showed that plasma sEV CLD3 has a good diagnostic value in distinguishing PCa patients with a Gleason score ≥ 8 from those with scores of 6–7 and benign prostatic hyperplasia (BPH) patients [137]. Park et al. recently demonstrated the potential use of sEV protein PSMA as a diagnostic marker in PCa [138]. Plasma/serum levels of STEAP1[140], CA IX [141], Del-1[143], and ephrin A2 [144] were also reported to distinguish between PCa patients and healthy individuals and/or BPH patients. Gamma-glutamyltransferase 1 was shown to be expressed at higher levels in urinary sEVs and tissues of PCa patients [145]. Zhang reported that urine sEV protein markers (SERPINA3, LRG1, and SCGB3A1) were higher in PCa patients (n = 20) than in healthy controls (n = 20), with SERPINA3 showing the highest correlation in distinguishing patients from healthy individuals [147]. Additionally, FABP5 was found to be not only higher in the PCa group compared with the control group, but also significantly correlated with Gleason grading in urinary sEVs [146].

sEV proteins are also used as non-invasive prognostic biomarkers of PCa. In a study conducted by Krishn et al., sEV αvβ3 integrin was demonstrated to be served as a non-invasive biomarker in the blood of PCa patients, and act as a molecule of intercellular communication that leads to disease progression [142]. Elevated expression of ACTN4 was found in the plasma sEVs of castration resistant prostate cancer (CRPC) patients and untreated PCa group, as well as in DU145 and PC3 cells. Downregulation of ACTN4 expression inhibited cancer cell growth, indicating that sEV ACTN4 may be a prognostic marker for evaluating tumor burden [136]. Increased expression of sEV PD-L1 was found to be associated with Radium-223 radiotherapy, indicating this sEV protein may aid in monitoring the response of PCa to radiation therapy [139].

Currently, the exploration of PCa-derived sEV proteins as biomarkers is still in infancy. Although these studies have shown promising results in screening or as diagnostic/prognostic biomarkers, further clinical translation faces significant obstacles. Firstly, there is a lack of large set of clinical samples or reliable cutoff values to evaluate its real clinical diagnostic values. Secondly, the potential sEV protein biomarkers identified need to be compared with traditional clinical diagnostic tools such as blood PSA test, MRI examination and tissue biopsies for sensitivity and specificity. Thirdly, there is lack of functional and mechanistic studies to investigate their roles in PCa progression and metastasis. If these issues can be addressed, and clinical challenges may be overcome, sEV proteins have the good potential to become new diagnostic tools for PCa diagnosis and prognosis in the future.

## sEV protein based potential therapy in prostate cancer

Due to the natural intercellular communication function, good biocompatibility, low immunogenicity, low toxicity, long blood circulation capability, biodegradability, and the ability to cross various biological barriers, sEVs have been emerging as a promising drug delivery approach in cancer treatment [148]. Due to the widespread adoption of sEV proteomics analysis, the types and functions of proteins derived from sEVs obtained from cell lines or bodily fluids are continuously being discovered. This has provided a new avenue of hope for utilizing sEV proteins in the treatment of cancer. Currently, there are three potential directions for cancer therapy based on sEVs proteins:

### Carrying therapeutic protein for targeted cancer therapy

Considerable efforts have been made to investigate the capacity of sEVs as carriers for cargo delivery while ensuring their toxicity towards cancer cells. sEVs have been a focus of research as carriers of chemotherapy drugs. It was shown that sEVs derived from PC3 cells increased the solubility of paclitaxel (PTX) in aqueous solutions compared to the control group of pure water, indicating their potential as a carrier [71]. Saari et al. discovered that sEVs carrying PTX entered LNCaP and PC3 cells through endocytosis and exerted cytotoxic effects on the cells. However, a new challenge has emerged: Certain types of sEVs without drugs can increase the vitality of cancer cells and reduce the cytotoxicity of PTX [149]. Furthermore, there were reports indicating that engineered sEVs, Exo Ce6+R848, created by co-cultivating the photosensitizer Chlorin e6 (Ce6), the immune adjuvant R848, and HEK 293 T cell-derived sEVs, synergistically converted M2 macrophages into M1 macrophages upon ultrasound irradiation, further activating effector T cells and creating an immune-promoting microenvironment [150]. Furthermore, a study discovered that the binding of serum-derived sEVs with TGFβRI kinase inhibitors or TLR7/8 agonists effectively inhibited the migration of PC3 cells through endocytosis. This binding also enhanced the release of pro-inflammatory cytokines from macrophages and DCs, ultimately leading to the occurrence of anti-tumor immune responses. The results of this study suggest that it is possible to achieve engineered sEV-based therapy for PCa in the future using TGFβRI kinase inhibitors and TLR7/8 agonists [151]. Recent research has found that sEV shows promising results and biocompatibility in the treatment of PCa. For example, sEVs derived from Akkermansia muciniphila when intravenously injected into PCa mouse Xenograft mode, resulted in an increased proportion of CD8^+^ T cells for granzyme B (GZMB) and interferon-gamma (IFN-γ) lymphocytes, leading to an increased M1 macrophages and a decreased M2 macrophages. Further in vitro experiments demonstrated that sEVs derived from Akkermansia muciniphila inhibited the proliferation and invasion of PCa cells [152]. PCa arises from a cancer stem or progenitor cell with homogeneous characteristics. Treatment with miR-let-7c loaded onto mesenchymal stem cell (MSC) sEVs resulted in a significant reduction in the proliferation and migration of CRPC-like PC3 and CWR22Rv1 cells, achieving a possibility of sEV therapy for CRPC [153]. These studies indicate that regardless of whether the sEVs are compatible with biomacromolecules or drugs, they demonstrate strong loading capacity. This lays the foundation for further research on their ability to carry therapeutic proteins and counteract the progression of PCa.

A study found that in a syngeneic mouse model, sEVs carrying the surface membrane protein SIRPα were transported to tumor sites, disrupting the CD47-SIRPα interaction and increasing the ability of macrophages to engulf tumor cells. This led to the inhibition of tumor growth. Additionally, it enhanced T cell infiltration and increased the possibility of releasing innate and adaptive anti-tumor responses through CD47-targeted therapy [154]. Currently, research on using sEV as carriers to deliver sEV proteins for the treatment of PCa is still relatively limited. The current research on sEV proteins as therapeutic carriers is still in the early stage. However, the aforementioned studies provide a new approach for PCa therapy. Regardless of the source of sEVs or the different endogenous contents and exogenous drugs they carry, they demonstrate a good ability to target tumor cells while ensuring sufficient safety and efficacy. The diversity of sEVs and their associated cargo set a solid foundation for personalized cancer treatment.

### sEV proteins as novel targets for therapy

sEVs can function as mediators of intercellular communication through paracrine or autocrine signaling. As mentioned earlier in this article, recent proteomic studies on sEV proteins in PCa cell lines, plasma, tissues, and other sources have revealed their involvement in sEV biogenesis, cargo loading, transport, and their subsequent impact on PCa cell development, progression, and prognosis. At each of these stages, sEV proteins play a crucial role. Therefore, the sEV proteins released by PCa cell lines, as depicted in Fig. 3, represent potential therapeutic targets. For instance, PD-L1, frequently overexpressed in PCa, poses challenges for immune checkpoint inhibitors due to the presence in the TME. In recent years, it has been discovered that tumor cells exert immune suppression by secreting sEVs carrying PD-L1, which binds to PD-1 on T cells. Consequently, this phenomenon has gained significant attention. One study found that Sulfisoxazole effectively inhibited plasma sEV PD-L1 levels in tumor-bearing mice, preventing CD8^+^ T cells from being depleted by PD-L1. This approach successfully overcame immune evasion mechanisms employed by cancer cells, offering a new therapeutic option for PCa treatment [155]. Furthermore, during the development of PCa, the role of immunosuppressive cells has been observed. M2 macrophages, commonly believed to communicate with tumor cells through paracrine signaling, were found to be associated with poorer prognosis and survival rates [156]. A study revealed that co-culturing M2 macrophages with PCa cells unveiled the M2 cells’ ability to enhance the NOTCH signaling, promoting PCa invasiveness. The intercellular communication mediated by M2 macrophages has received significant attention [157]. In a recent study by Cui et al. co-culturing M2 macrophages and colon cancer cells demonstrated that sEV protein ferritin heavy chain (FTH1) was transferred from M2 macrophages to colon cancer cells, promoting their proliferation. This finding highlights the importance of sEVs as essential mediators in intercellular communication. The sEV target protein FTH1 on M2 macrophages provides us with a potential therapeutic approach [158]. In a recent study using a preclinical tumor model, researchers discovered that sEVs derived from M2 macrophages downregulated tumor cell MHC-I expression through the delivery of apolipoprotein E (ApoE). This suppression inhibited tumor intrinsic immunogenicity, ultimately leading to resistance to immune checkpoint blockade (ICB). However, the ApoE ligand EZ-482 reversed the M2-sEV-induced ICB resistance. The results of this study suggest that ApoE ligand EZ-482 could be beneficial for tumor patients, particularly those with M2-rich tumors such as PCa, providing a theoretical basis for combined immunotherapy with ICB.

### Modifications of sEV proteins

Current research primarily focuses on modifying sEV proteins to develop sEV vaccines and enhance the loading and delivery capacity of sEV-mediated biomolecular cargo. Sipuleucel-T, an FDA-approved cancer vaccine targeting prostate acid phosphatase (PAP) antigen in PCa, consists of autologous peripheral blood mononuclear cells enriched with autologous DCs and has shown the survival benefits in patients with CRPC [159]. Studies have found that sEVs derived from DCs activate CD4^+^ and CD8^+^ T cells, triggering anti-tumor immune responses [160]. Furthermore, the composition and function of sEVs can be altered by adjusting cell types, isolation, and purification conditions to meet the personalized treatment demands of vaccines. Therefore, researchers are exploring the use of sEVs as vaccines for PCa treatment. As early as 2011, a high attenuated vaccine called MVA-BN was selectively engineered to incorporate PSA or PAP. Targeting of sEVs was achieved by fusing the antigens to the milk fat globule-EGF factor 8 (MFG-E8) C1C2 domain. Results from treating PCa mouse models with these vaccines showed that the group treated with MVA-BN-PSA-C1C2 exhibited increased immunogenicity against PSA compared to the control group, and it improved the efficacy of anti-tumor therapy [161]. Shi et al. anchored IFN-γ fusion protein to the surface of sEVs derived from PCa cells to develop a novel sEV vaccine. This vaccine demonstrated the ability to increase the population of M1 macrophages in the body and downregulate the expression of vascular endothelial growth factor receptor 2 (VEGFR2), further induced the production of specific antibodies against sEVs which weakened the pro-tumorigenic effects of PCDEVs, and inhibited tumor growth [162]. The development of tumor vaccines based on sEVs still faces many challenges. For example, cold tumors like PCa have a unique tumor immune-suppressive microenvironment, making them insensitive to immunotherapy [163]. Additionally, they exhibit different antigen expression patterns [164], raising concerns about whether sEV-based vaccines can effectively cover antigen variations. Furthermore, there are difficulties in production, optimization of delivery, and determining the therapeutic efficacy and dosage, all of which are inevitable obstacles. Nevertheless, understanding the immune evasion mechanisms of PCa is our primary goal in developing sEV-based vaccines. Since the discovery that functional sEV RNAs and proteins can mediate communication between EVs and tumor cells, there has been great interest in understanding how bioactive molecules are loaded into sEVs and delivered to tumor cells.

Understanding the regulatory mechanisms that influence loading and delivery efficiency is crucial to harnessing the full potential of sEVs as a tool for tumor treatment. Current research reveals the involvement of sEV proteins in these processes, suggesting that modulation of loading and delivery can be achieved through modifications of sEV proteins. Es-Haghi et al. developed a fusion protein (hCD9.hAGO2) that combines sEV membrane protein CD9 with RNA-binding protein AGO2. Compared to sEVs isolated solely by overexpressing the desired miRNA or shRNA, this fusion protein exhibited a higher level of miRNA and shRNA loading capacity and was effectively delivered to recipient cells [165]. Another study utilized a targeted and modular EV loading (TAMEL) system, which involved fusing the MS2 bacteriophage capsid protein with sEV-associated proteins. This approach resulted in a six-fold increase in RNA loading compared to the control group. However, when this system was applied for delivery to PC3 cells, it was found that this modification reduced the RNA payload in sEVs and restricted their release in the cytoplasm. This could be due to the modification altering the subcellular localization, thereby affecting the interaction between sEVs and recipient cells and limiting endosomal escape. This suggests the need for further optimization of the modification strategy to gradually enhance the cargo RNA loading capacity and release efficiency [166].

All these studies indicate that sEVs are promising drug delivery candidates, although their use also has some limitations, such as insufficient targeting ability, which limits their clinical application as therapeutic carriers. Currently, researchers are exploring ways to enhance the natural targeting abilities of sEVs through modifications. This involves modifying sEVs to improve their existing targeting capabilities or to enable them to target cells that they normally wouldn’t. One promising application of such modifications is the construction of a new type of cell-derived, surface-modified liposome carrier for nanodrug therapy. For example, Pan et al. developed a nanovector called Exo-PMA/Fe-HSA@DOX that was able to block the EGFR/AKT/NF-kB/IkB signaling pathway and achieve targeted therapy for PCa [167]. Altanerova et al. showed that MSCs-Exo modified with superparamagnetic iron oxide nanoparticles and exposed to an external alternating magnetic field induced toxicity in PC3 cells [168]. More recently, a novel nano-platform that mimics sEVs has been developed for loading tumor therapeutic drugs. This platform exhibits similar drug loading capacity and targeting ability for tumor cells as nature sEVs but is more controllable and productive [169]. Currently, there have been studies on the generation of sEV-mimetic nanovesicles from standardized MSCs derived from human induced pluripotent stem cells (iPSCs). A comparison between these nanovesicles carrying docetaxel and free docetaxel in a mouse model of PCa revealed that the docetaxel-loaded sEV-mimetic nanovesicles exhibited enhanced tumor targeting ability and significantly suppressed tumor growth [170]. In addition, Severic et al. engineered prostate specific membrane antigen (PSMA)-targeting peptides onto the surface of sEV mimetics, which effectively targeted PSMA-positive PCa cell lines (specifically LNCaP and C4-2B). These engineered vesicles exhibited excellent targeting ability in both in vitro and in vivo experiments [171].

**Fig. 5:**
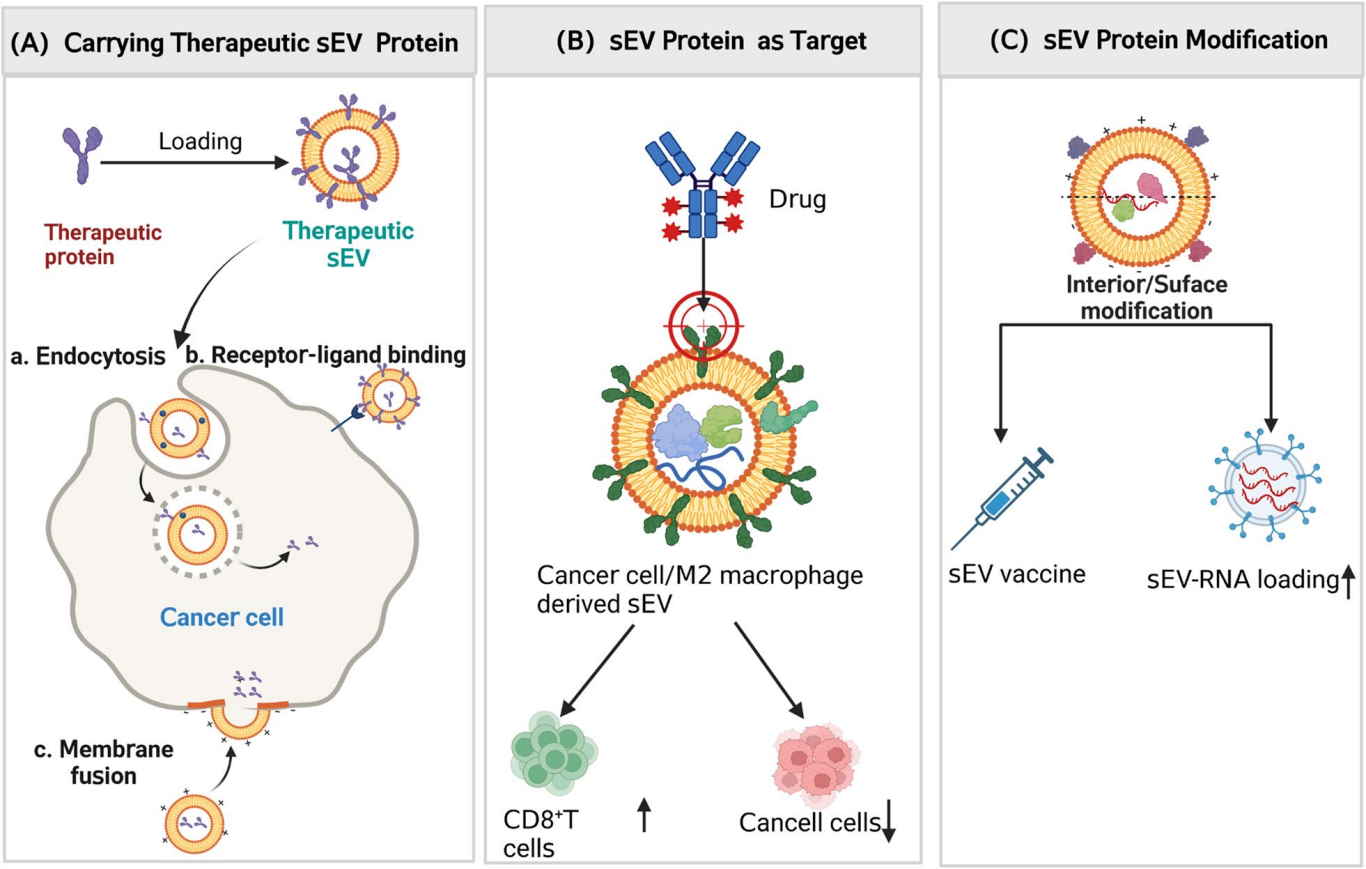
Three potential sEV protein-based prostate cancer treatments. **A** sEVs exert cytotoxic effects by delivering therapeutic sEV proteins to cancer cells. **B** Cancer-derived sEV proteins serve as therapeutic targets. **C** Modification of sEV proteins can be employed to develop vaccines and enhance the payload capacity of sEV-RNA This figure was created with BioRender.com

Overall, modifying sEVs holds great potential for advancing nanodrug therapy. Although endogenous delivery vehicles, including sEVs, are safe and efficient drug delivery system (DDS), they still face significant challenges, such as the unfavorable characteristics of sEVs, low volume, high heterogeneity, complex cargo, and difficulties in characterization, which may hinder their clinical translation. Figure 5 illustrates three current potential sEV proteins-based PCa treatments.

## Conclusions and perspectives

PCa-derived proteins are important for intercellular communication and transport, and they play a critical role in the progression of the disease. As we gain a deeper understanding of the roles and signaling pathways mediated by sEV proteins, we can further elucidate the mechanisms underlying PCa progression. sEV proteins are stable and present in much higher quantities in patients than in healthy individuals, making them a promising biomarker for diagnosing and prognosing PCa progression and treatment response. The EMT mediates changes in multiple steps during PCa deterioration, highlighting the need to identify common upstream pathways that regulate sEV protein mediated EMT. Developing sEV-based therapies that target multiple pathways may be necessary. sEV proteins participate in PCa metastasis through multiple mechanisms, including promoting tumor cell growth and proliferation, inducing angiogenesis abnormalities, facilitating EMT, remodeling the TME, and facilitating PMN formation. Understanding the specific roles of these sEV proteins in PCa metastasis may help us identify therapeutic targets and ultimately inhibit their pro-metastatic functions, thereby facilitating the development of precision treatment strategies based on sEV proteins.

PD-1/PD-L1 are crucial negative co-stimulatory molecules in PCa that mediate tumor immune evasion, and their role has been validated in sEVs. Their expression also increases after radiotherapy, suggesting that developing combination therapies with sEV immune checkpoint inhibitors is a promising new approach. In this review, we innovatively summarize three main therapeutic strategies of sEV proteins in PCa. sEV proteins provide insights into the mechanisms of sEV biogenesis, protein–protein interactions, and receptor targeting, offering therapeutic potential. sEV protein-based therapy may provide a new avenue for the treatment of ENPC and CRPC. However, unfortunately, the application of sEV protein-based therapy in PCa is not yet widespread. This is primarily due to the lack of protein carrier amplification procedures in sEV proteomics analysis, which adds clinical complexity. Additionally, the immunosuppressive microenvironment in PCa hinders the development of immunotherapy. Currently, all sEV protein-based treatments involve the immune microenvironment composed of immune cells and immune suppressor cells.

Overall, sEV proteins play a pivotal role in prostate carcinogenesis, and have the great potential to be used as diagnostic, prognostic, and therapeutic agents in PCa. The emergence of single-vesicle analysis allows us to explore the heterogeneity and functions of different subpopulations of sEV and their cargo proteins in the TME. It has made significant contributions in revealing the diversity of EV subpopulations and their protein content, as well as their potential roles in the progression and treatment of PCa. While progress has been made, there is still a long way to go for the translation of sEVs from laboratory research to clinical applications. It is important to acknowledge that the lack of standardized techniques for sEV isolation and characterization is a significant limitation, and large-scale prospective studies are necessary to validate the diagnostic accuracy of sEV proteins. Despite these challenges, we believe that the value of sEV proteins in the field of cancer will be realized in the near future with the establishment of standardized processes from production to effective application.

## Data Availability

Not applicable.
